# Ten-year distant-recurrence risk prediction in breast cancer by CanAssist Breast (CAB) in Dutch sub-cohort of the randomized TEAM trial

**DOI:** 10.1186/s13058-023-01643-2

**Published:** 2023-04-14

**Authors:** Xi Zhang, Aparna Gunda, Elma Meershoek-Klein Kranenbarg, Gerrit-Jan Liefers, Badada Ananthamurthy Savitha, Payal Shrivastava, Chandra Prakash Vijay Kumar Serkad, Taranjot Kaur, Mallikarjuna Siraganahalli Eshwaraiah, Rob A. E. M. Tollenaar, Cornelis J. H. van de Velde, Caroline M. J. Seynaeve, Manjiri Bakre, Peter J. K. Kuppen

**Affiliations:** 1 Department of Surgery, Leiden University Medical Center (LUMC), Albinusdreef 2, Leiden, 2333 ZA The Netherlands; 2 OncoStem Diagnostics Pvt Ltd, #4, Raja Ram Mohan Roy Road, Aanand Tower, 2nd Floor, Bangalore, 560027 India; 3 Geriatric Oncology Research Group, Leiden University Medical Center (LUMC), Leiden, The Netherlands; 4 Medical Oncology, Erasmus Medical Center, Rotterdam, The Netherlands

**Keywords:** CanAssist Breast (CAB), Early breast cancer, Adjuvant, Endocrine therapy, Distant recurrence

## Abstract

**Background:**

Hormone receptor (HR)-positive, HER2/neu-negative breast cancers have a sustained risk of recurrence up to 20 years from diagnosis. TEAM (Tamoxifen, Exemestane Adjuvant Multinational) is a large, multi-country, phase III trial that randomized 9776 women for the use of hormonal therapy. Of these 2754 were Dutch patients. The current study aims for the first time to correlate the ten-year clinical outcomes with predictions by CanAssist Breast (CAB)—a prognostic test developed in South East Asia, on a Dutch sub-cohort that participated in the TEAM. The total Dutch TEAM cohort and the current Dutch sub-cohort were almost similar with respect to patient age and tumor anatomical features.

**Methods:**

Of the 2754 patients from the Netherlands, which are part of the original TEAM trial, 592 patients’ samples were available with Leiden University Medical Center (LUMC). The risk stratification of CAB was correlated with outcomes of patients using logistic regression approaches entailing Kaplan–Meier survival curves, univariate and multivariate cox-regression hazards model. We used hazard ratios (HRs), the cumulative incidence of distant metastasis/death due to breast cancer (DM), and distant recurrence-free interval (DRFi) for assessment.

**Results:**

Out of 433 patients finally included, the majority, 68.4% had lymph node-positive disease, while only a minority received chemotherapy (20.8%) in addition to endocrine therapy. CAB stratified 67.5% of the total cohort as low-risk [DM = 11.5% (95% CI, 7.6–15.2)] and 32.5% as high-risk [DM = 30.2% (95% CI, 21.9–37.6)] with an HR of 2.90 (95% CI, 1.75–4.80; *P* < 0.001) at ten years. CAB risk score was an independent prognostic factor in the consideration of clinical parameters in multivariate analysis. At ten years, CAB high-risk had the worst DRFi of 69.8%, CAB low-risk in the exemestane monotherapy arm had the best DRFi of 92.7% [*vs* CAB high-risk, HR, 0.21 (95% CI, 0.11–0.43), *P* < 0.001], and CAB low-risk in the sequential arm had a DRFi of 84.2% [*vs* CAB high-risk, HR, 0.48 (95% CI, 0.28–0.82), *P* = 0.009].

**Conclusions:**

Cost-effective CAB is a statistically robust prognostic and predictive tool for ten-year DM for postmenopausal women with HR+/HER2−, early breast cancer. CAB low-risk patients who received exemestane monotherapy had an excellent ten-year DRFi.

**Supplementary Information:**

The online version contains supplementary material available at 10.1186/s13058-023-01643-2.

## Background

Nowadays, integrated models are used to make treatment decisions about escalation or de-escalation of either endocrine therapy or chemotherapy for early-stage, hormonal receptor (HR)-positive, *HER2/neu*-negative breast cancer, with the consideration of both anatomical and biologic risk [1]. Genomic tools, such as the 21-gene recurrence score (RS, Oncotype-Dx), 70-gene recurrence score (MammaPrint), PAM50 (Prosigna), and EndoPredict Clin (EPClin) provide clinicians with information on clinical prognosis or chemotherapy response prediction [2–5].

However, there is a great need for better prognostic/predictive tests in HR+/HER2−breast cancer that dig deeper into tumor biology independent of proliferative capacity, including aggressive biology of younger or node-positive patients. Also, prognostic tools useful in long-term risk predictions in patients with clinically intermediate risk (we defined it as one to three lymph nodes involved and/or grade II, TNM stage II) diseases are still elusive and will undoubtedly add value. Current genomic tools are based on either qPCR or RNA microarray methods, which are expensive and may increase the social-economic burden, some of them without combining clinical parameters. Immunohistochemistry-based tests have several advantages, such as direct and functional state visualization of proteins, quantitative and cellular location information, and not being confounded by effects of non-tumorous tissues.

CanAssist Breast (CAB), a prognostic test was developed based on three clinical parameters (tumor size, grade, and axillary lymph nodes) and the expression of five biomarkers (CD44, N-Cadherin, pan Cadherin, ABCC4 and ABCC11) quantitated using immunohistochemistry and scoring by pathologists [6]. CAB uses a Support Vector Machine Learning (SVML)-based algorithm to predict risk score and category (high or low) [7–9]. CAB predicts the risk of distant recurrence within five years from diagnosis in early-stage, HR+/HER2− breast cancer patients. CAB has been validated on breast cancer patient cohorts from India, the USA, and Europe showcasing identical performance on 5-year recurrence risk predictions across multiple races/ethnicities [8, 10, 11]. It was interesting to see 83–85% concordance in the low-risk group between CAB and Oncotype DX/ MammaPrint, the highest ever shown between any two prognostic tests [11, 12]. Interestingly, CAB stratified a higher percentage of the previous retrospective cohort as low-risk than the IHC4 score and re-stratified over 74% of patients with both high Ki-67 and IHC4 intermediate-risk score into the low-risk category [13]. Nevertheless, it has not been validated in the context of a phase III randomized trial and moreover, it would be judicious to evaluate the performance of CAB for long-term recurrence risk predictions.

Therefore, the present exploration analysis is sought to clinically validate CAB in the Dutch sub-cohort of the TEAM trial (Tamoxifen, Exemestane Adjuvant Multinational). TEAM trial showed that disease-free survival was similar between the two arms (exemestane alone versus sequential treatment with tamoxifen followed by exemestane) at ten years [10, 14, 15]; thus it requires an additional assay to further differentiate the prognosis of these two arms. The current validation study on tumor samples from the TEAM trial aimed to assess the ability of CAB to predict the risk of distant recurrence at ten years for the first time, for postmenopausal patients with early breast cancer, HR-positive, HER2-negative, node-negative or positive disease.

## Methods

### Study population

The parent trial for this study was TEAM, a global multicenter, open-label, randomized, phase III study of postmenopausal women with HR+breast cancer, conducted from 2002 to 2016 (Trial Registration: ClinicalTrials.gov NCT00279448, NCT00032136, and NCT00036270; Netherlands Trial Registry NTR267). The inclusion and exclusion criteria and registration information of TEAM were described previously [10]. Patients were randomly assigned (1:1) to receive exemestane (25 mg once a day, orally) or sequential therapy (tamoxifen of 20 mg once a day, orally for 2.5–3.0 years, and switched to exemestane therapy for a total duration of 5.0 years). Stratification factors included center, adjuvant chemotherapy (yes vs no) and time period between surgery and starting hormonal therapy (0–3 months vs 3–6 months vs > 6 months). In the original TEAM trial, 6120 patients were involved in the intention-to-treatment (ITT) analysis. The samples from a cohort of 592 patients were collected from the Dutch trial population of 2754 patients. Finally, CAB was successfully done on 480 patient samples. Patients with HER2-positive receptor status, TNM stage IV or missing lymph node status, and loss of follow-up were excluded from the final analysis of the present study (Additional file 1: Figure S1). The study has been carried out in accordance with the Declaration of Helsinki. All patients provided informed consent.

### Study end points

The primary endpoint of this study was the cumulative incidence of distant recurrence or metastasis/death due to breast cancer (DM), or the freedom from distant metastasis or death due to breast cancer (known as [DRFi]). DRFi was defined as the time from randomization to distant recurrence or death due to breast cancer if no recurrence was reported before death. Other endpoints were cumulative incidence of relapse or recurrence-free survival (RFS), cumulative mortality or overall survival (OS), and cumulative incidence of invasive breast cancer disease or invasive disease-recurrent free survival (iDFS). RFS was defined as the time from randomization to any breast cancer recurrence or death due to breast cancer if no recurrence was reported before death. OS was defined as the time from randomization to death due to any cause. iDFS was defined as the time from randomization to disease recurrence (locoregional or distant recurrence or new primary breast cancer, excluding ductal carcinoma in situ [DCIS] of any kind and new second primary non-breast cancer) or death from any cause.

### Statistical analysis

Kaplan–Meier estimates of DM, cumulative incidence of relapse, cumulative mortality, and cumulative incidence of invasive disease were calculated for CAB risk groups for the total cohort, treatment groups or designated subgroups, compared by log-rank tests. Univariate and multivariate hazard ratios (HRs), as well as the interaction between subgroups and prognostic factors, were calculated by the Cox proportional hazard model. For multiple comparisons, the *P* value was adjusted using the method of Benjamini–Hochberg (BH). Data were collected in the Central Statistical and Data Centre (Leiden University Medical Center, LUMC, Leiden, the Netherlands). Statistical analyses were done with R (4.0.3 version) with the Survival, Survminer, Ggplot2, Ggpubr, Forestplot, Rms, and Rstatix packages. The endpoints were censored at 10.0 years. All tests were two-sided, and a *P*-value of less than or equal to 0.05 was considered significant.

### Performance of CAB

Tumor samples were initially assessed for amount of tumor with the hematoxylin and eosin staining method. CAB is only performed in OncoStem’s CAP (College of American Pathologists) and ISO13485 accredited laboratory in Bangalore, India. Tumor samples with greater than 30% of tumor content were processed for immunohistochemistry (IHC) experiments for CAB markers. IHCs were performed on a Ventana Benchmark automated IHC stainer [8]. The IHC grading information along with clinical parameters (tumor size, histological grade, node status) was provided as inputs to the statistical algorithm. The algorithm predicts a risk score and based on the predefined threshold, patients with a risk score of below and equal to 15.5 are deemed to have a low risk for distant recurrence and patients with a risk score of above 15.5, are considered as a high risk for distant recurrence [16]. The team performing CAB were blinded to patient outcomes. 

## Results

### Patient and tumor characteristics

Of the 592 Dutch patients enrolled in TEAM, 433 (73.1%), consistent with the inclusion criteria of the present study, underwent CanAssist Breast (CAB) test (Additional file 1: Figure S1). The two trial arms in the current study were well-balanced, with a median follow-up of 10.5 years (IQR 10.4–10.6). A total of 221 patients were randomized to the exemestane arm, and 212 patients were randomized into the sequential arm (Table 1). The median age was 65.0 years. The majority were T2 (51.3%), lymph node-positive (68.4%), TNM stage II (79.4%), and grade II-III (79.5%). Most of the patients (79.2%) received endocrine therapy alone. 404 (93.3%) patients had lymph node stage of pN0-1, defined as node-negative (31.6%) plus one to three nodes (61.7%) involved, while 6.7% had N2 disease. The distributions of the tumor stage, lymph node stage, TNM stage, age, and estrogen receptor (ER) were not significantly different compared to the Dutch population (*n* = 2754 patients), while in the current study, there were higher proportions of low-grade, progesterone receptor (PR)-positive and patients without receiving chemotherapy (Table 1).

**Table 1:** Baseline patient characteristics

	Exemestane Arm (*N* = 221)	Sequential Arm (*N* = 212)	Total (*N* = 433)	Dutch population in TEAM trial analysis (*N* = 2754)^a^	*P* value (current study *vs*. Dutch population in the TEAM trial)^b^
Age					
Median [Min, Max]	65.0 [46.0, 85.0]	64.5 [46.0, 90.0]	65.0 [46.0, 90.0]	64.0 [38.0, 96.0]	0.694
Tumor stage					
pT1	100 (45.2%)	94 (44.3%)	194 (44.8%)	1236 (44.9%)	0.423
pT2	112 (50.7%)	110 (51.9%)	222 (51.3%)	1329 (48.3%)	
pT3	5 (2.3%)	3 (1.4%)	8 (1.8%)	120 (4.4%)	
pT4	4 (1.8%)	5 (2.4%)	9 (2.1%)	63 (2.3%)	
Lymph node stage					
pN0	71 (32.1%)	66 (31.1%)	137 (31.6%)	832 (30.2%)	0.085
pN1	139 (62.9%)	128 (60.3%)	267 (61.7%)	1454 (52.8%)	
pN2	11 (5.0%)	18 (8.5%)	29 (6.7%)	327 (11.9%)	
TNM stage					
I	30 (13.6%)	22 (10.4%)	52 (12.0%)	354 (12.9%)	0.208
IIA	102 (46.2%)	105 (49.5%)	207 (47.8%)	1183 (43.0%)	
IIB	74 (33.5%)	63 (29.7%)	137 (31.6%)	661 (24.0%)	
IIIA	11 (5.0%)	17 (8.0%)	28 (6.5%)	352 (12.8%)	
IIIB	4 (1.8%)	5 (2.4%)	9 (2.1%)	53 (1.9%)	
Histological grade					
G1	40 (18.1%)	49 (23.1%)	89 (20.6%)	420 (15.3%)	< 0.001
G2	117 (52.9%)	115 (54.2%)	232 (53.6%)	1219 (44.3%)	
G3	64 (29.0%)	48 (22.6%)	112 (25.9%)	934 (33.9%)	
Estrogen receptor (ER)					
Positive	219 (99.1%)	209 (98.6%)	428 (98.8%)	2700 (98.0%)	0.335
Negative	2 (0.9%)	3 (1.4%)	5 (1.2%)	53 (1.9%)	
Progesterone receptor (PR)					
Positive	185 (83.7%)	176 (83.0%)	361 (83.4%)	1999 (72.6%)	< 0.001
Negative	35 (15.8%)	31 (14.6%)	66 (15.2%)	601 (21.8%)	
Not determined	1 (0.5%)	5 (2.4%)	6 (1.4%)	153 (5.6%)	
Chemotherapy received					
Yes	44 (19.9%)	46 (21.7%)	90 (20.8%)	812 (29.5%)	< 0.001
No	177 (80.1%)	166 (78.3%)	343 (79.2%)	1942 (70.5%)	

^a ^For the tumor stage in the total Dutch population, there was one case classified as T0 or T in situ, not shown but calculated in this table. There were another five cases classified as Tx or unknown, not shown but calculated in this table. For the lymph node stage in the total Dutch population, 132 cases included N3, one case unknown, not shown but calculated in this table. For the TNM stage in the total Dutch population, there were 136 cases classified as IIIC or IV stage, 15 cases unknown, not shown but calculated in this table. For grade in the total Dutch population, there were 181 cases classified as grade unknown, not shown but calculated in this table. For ER in the total Dutch population, there was one case classified as unknown, not shown but calculated in this table. For PR in the total Dutch population, there was one case classified as unknown, not shown but calculated in this table. ^b^ For continuous variables with normal distribution, a t-test was performed. Chi-squared test was performed when comparing categories. For the trend in proportion, the Cochran-Armitage trend test was performed

### Validation of CAB in the Dutch cohort of TEAM trial

**Fig. 1:**
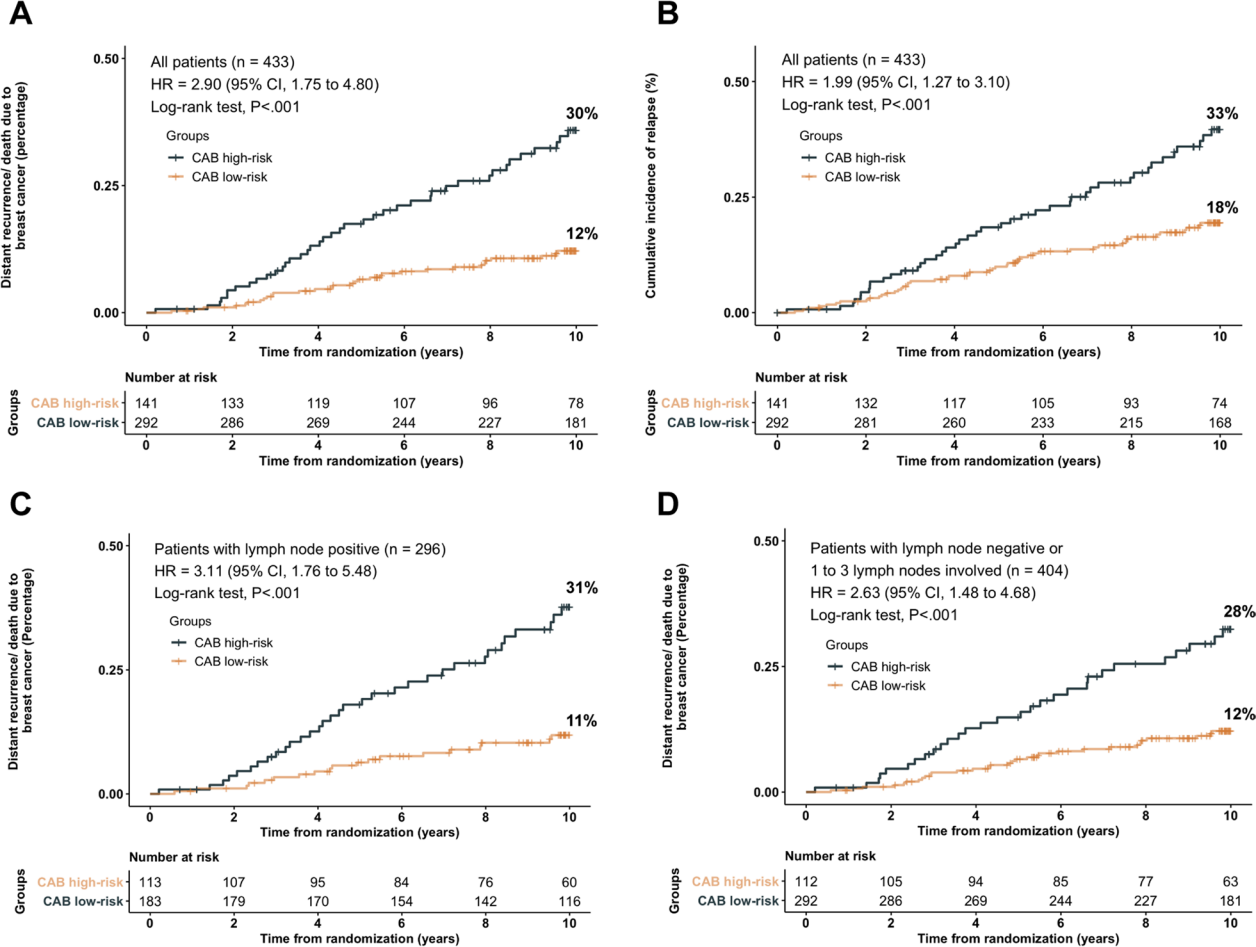
Cumulative incidence of distant recurrence/death due to breast cancer (DM), or cumulative incidence of relapse, stratified by CanAssist Breast (CAB) at ten years for the total cohort or subgroups. **A** DM for the total cohort. **B** Cumulative incidence of relapse for the total cohort. **C** Lymph node-positive subgroup. **D** Lymph node stage N0-1 subgroup

CAB was analyzed as a categorical variable (low or high-risk). 32.5% of patients were classified as the high-risk group, while the rest of them were in the low-risk group. In Kaplan–Meier survival analysis, CAB significantly stratified patients for the primary endpoint, DM [11.5% (95% CI, 7.6–15.2) for low-risk, *vs* 30.2% (95% CI, 21.9–37.6) for high risk; HR, 2.90 (95% CI, 1.75–4.80); *P* < 0.001; Fig. 1A]. Other endpoints included cumulative incidence of relapse (HR, 1.99; 95% CI, 1.27–3.10; *P* < 0.001; Fig. 1B), cumulative mortality (HR, 1.38; 95% CI, 0.93–2.07; *P* = 0.09; Additional file 2: Figure S2A), and cumulative incidence of invasive breast cancer recurrence (HR, 1.40; 95% CI, 0.98–2.01; *P* = 0.05; Additional file 2: Figure S2B). In each of the endpoints barring cumulative mortality, CAB high-risk patients had significantly higher recurrences/worse outcomes than CAB low-risk patients.

The Cox regression model was adjusted for T stage, lymph nodal status, grade, and CAB risk in multivariate analysis. The result showed that CAB risk score (HR, 2.54; 95% CI, 1.67–3.85; *P* < 0.001) was an independent prognostic factor for DM (Table 2).

**Table 2:** Multivariate analysis of Dutch-cohort of TEAM for distant metastasis or death due to breast cancer (DM)

Variable	DM ^a,c,d^ (total cohort)	
	Hazard ratio (95% CI)	*P* value
Tumor stage		
T1	Reference	–
T2-4	1.28 (0.85–1.93)	0.31
Lymph node status		
Negative	Reference	–
Positive	1.19 (0.75–1.88)	0.54
Histological grade		
G1	Reference	
G2 + 3	2.07 (1.00–4.27)	0.10
CAB risk category		
Low-risk	Reference	–
High-risk	2.54 (1.67–3.85)	< 0.001 ^b^

^a^Model undergone multivariate step-cox regression (both direction), adjusted for T stage (T2-4 vs T1), lymph nodal status (N+ vs N−), grade (G2 + 3 vs G1), and CAB risk score. Likelihood ratio was used to test whether the model was statistically significant. DM: distant metastasis or death due to breast cancer, CAB: CanAssist Breast. ^b^ *P* < 0.05. ^c^ Model likelihood ratio test = 24.59, *P* < 0.001. ^d^ There was only one patient who died due to breast cancer with only local recurrence, without distant metastasis

### CAB risk stratification in N+ and N0-1 subgroups

The CAB risk score was prognostic for DM in the subset of patients with lymph node-positive (N+; HR, 3.11; 95% CI, 1.76–5.48; *P* < 0.001; Fig. 1C), or in the subset of patients with N0-1 (HR, 2.63; 95% CI, 1.48–4.68; *P* < 0.001; Fig. 1D). During the first five years, CAB was also prognostic for early DM in N+ patients (HR, 2.60; 95% CI, 1.24–5.46; *P* = 0.008; Additional file 3: Figure S3A). The significant interaction between node status and CAB was recorded (*P*_*interation*_ < 0.001), but node status was not an independent risk factor in the multivariate regression model.

### Association of endocrine therapy and CAB sub-categories with survival outcomes

**Fig. 2:**
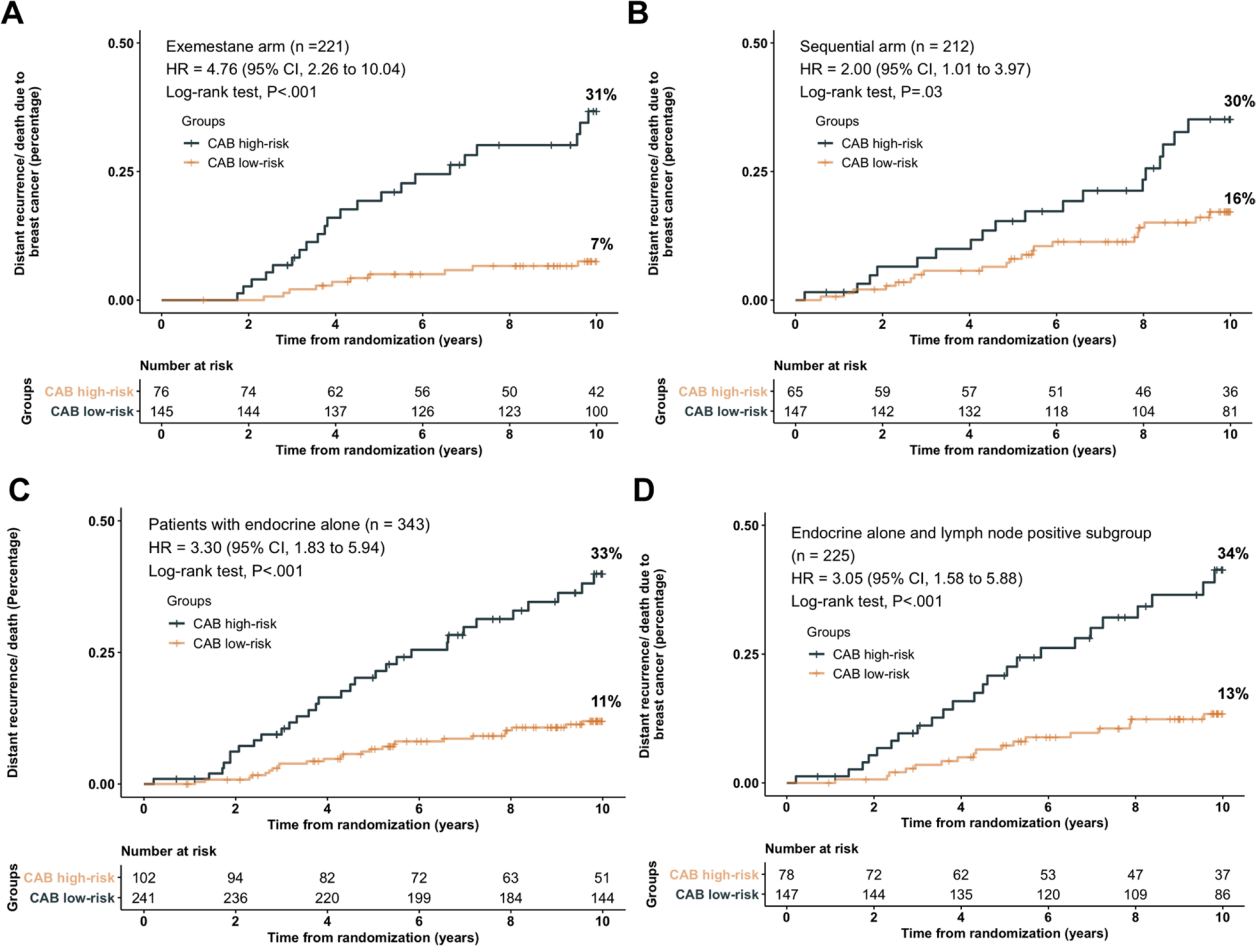
DM stratified by CAB in designated subgroups. **A** DM for exemestane arm. Interaction (randomization vs CAB) *P* < 0.001. **B** DM for the sequential arm. **C** Patients received endocrine therapy alone subgroup. **D** Patients with lymph node-positive received endocrine therapy alone

In the exemestane arm, patients were significantly classified into low or high-risk groups by CAB for DM (HR, 4.76; 95% CI, 2.26–10.04; *P* < 0.001; Fig. 2A). In the sequential arm, CAB could also predict the prognosis for DM (HR, 2.00; 95% CI, 1.01–3.97; *P* = 0.03; Fig. 2B). The stratification ability of CAB was better in the exemestane arm than in the sequential arm.

For patients with N+, there was significant association between CAB risk category and DM at ten years, in exemestane arm (HR, 4.74; 95% CI, 2.03–11.03; *P* < 0.001; Additional file 3: Figure S3B) and sequential arm (HR, 2.17; 95% CI, 1.01–4.65; *P* = 0.04; Additional file 3: Figure S3C).

### Prognostication by CAB in patients treated with endocrine therapy alone

CAB provided statistically significant prognostic information on DM in patients who received endocrine therapy alone (HR, 3.30; 95% CI 1.83–5.94; *P* < 0.001; Fig. 2C). For patients with N+ who did not receive chemotherapy, CAB could significantly stratify patients for DM as well (HR, 3.05; 95% CI, 1.58–5.88; *P* < 0.001; Fig. 2D). Furthermore, CAB could stratify patients into low or high-risk groups for DM in both arms; exemestane alone (HR, 4.59; 95% CI, 2.11–10.00; *P* < 0.001; Additional file 2: Figure S2C) or sequential regimen (HR, 2.32; 95% CI, 0.94–5.71; *P* = 0.03; Additional file 2: Figure S2D).

### Overall performance and predictive ability of CAB

**Fig. 3:**
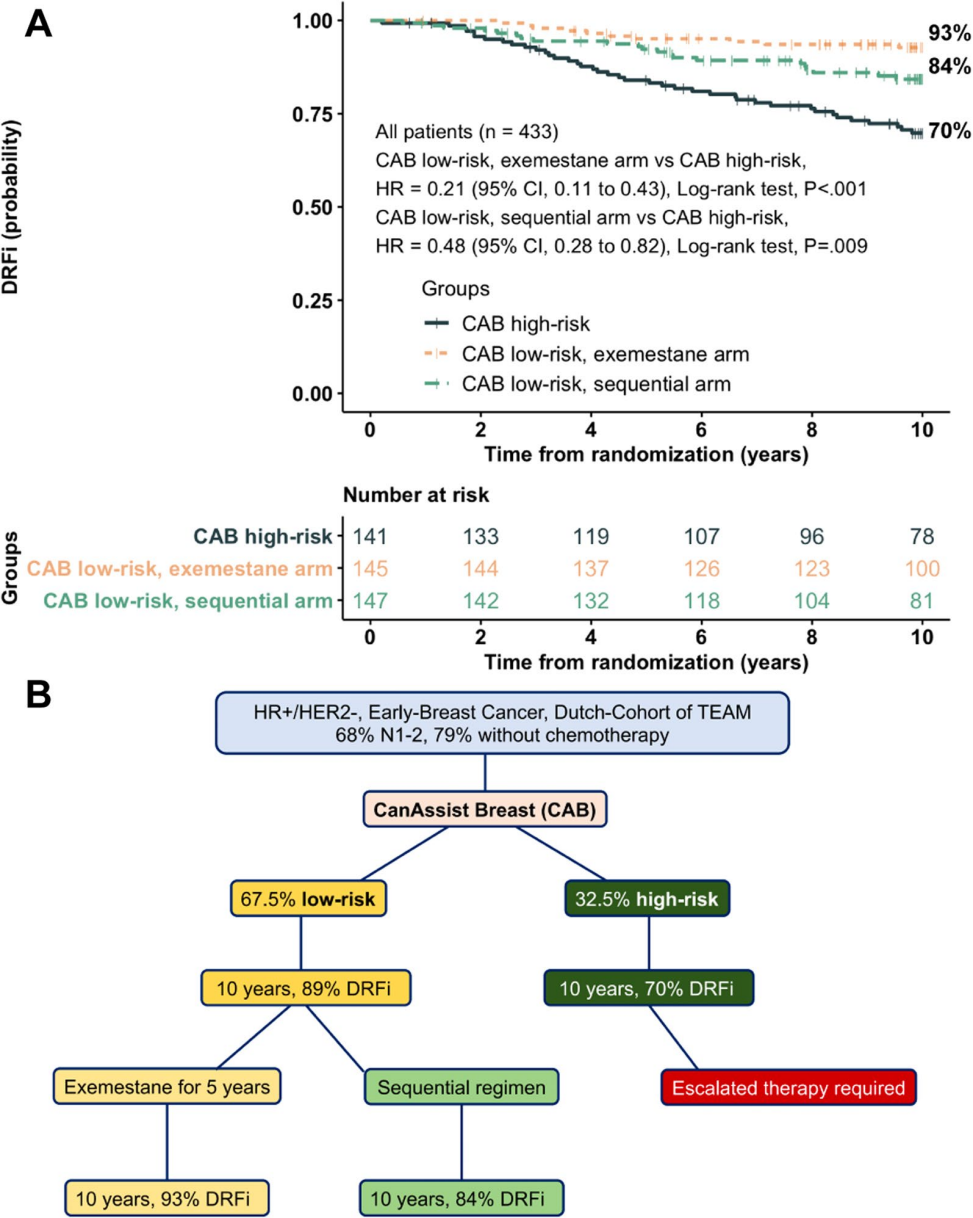
Overall CAB performance with prognostic and predictive values in HR+/HER2− early breast cancer patients. **A** DRFi was stratified into three groups: CAB high-risk, CAB low-risk in the exemestane arm, and CAB low-risk in the sequential arm. *P* value was adjusted using the method of Benjamini-Hochberg (BH). **B** Schematic overall performance of CAB

CAB statistically significantly stratified three groups at ten years (log-rank *P* < 0.001; Fig. 3A), including CAB high-risk with a DRFi of 69.8% (95% CI, 62.4–78.1), CAB low-risk in the exemestane arm with a DRFi of 92.7% [95% CI, 88.5–97.2; *vs* CAB high-risk, HR, 0.21 (95% CI, 0.11–0.43), *P* < 0.001], and CAB low-risk in the sequential arm with a DRFi of 84.2% [95% CI, 78.2–90.7; *vs* CAB high-risk, HR, 0.48 (95% CI, 0.28–0.82), *P* = 0.009].

In CAB low-risk group (Additional file 4: Figure S4A), for patients who received endocrine therapy alone, exemestane arm had better overall survival (OS; HR, 1.79; 95% CI, 1.16–2.78; *P* = 0.03), iDFS (HR, 1.92; 95% CI, 1.29–2.87; *P* = 0.007), and RFS (HR, 2.05; 95% CI, 1.17–3.58; *P* = 0.04) than the sequential arm, but this survival benefit was not observed for DRFi (HR, 1.51; 95% CI, 0.77–2.96; *P* = 0.31). In addition, in low-risk group (Additional file 4: Figure S4A), patients who received chemo-endocrine therapy did not have an advantage of improved survival benefit for either OS (HR, 1.99; 95% CI, 1.03–3.83; *P* = 0.09), iDFS (HR, 1.42; 95% CI, 0.85–2.38; *P* = 0.24), RFS (HR, 0.99; 95% CI, 0.54–1.82; *P* = 0.98), or DRFi (HR, 0.93; 95% CI, 0.44–1.97; *P* = 0.88), when compared with endocrine therapy alone.

In high-risk group, for patients who received endocrine therapy alone (Additional file 4: Figure S4B), there was no significant difference in the outcomes between two randomized arms for all the end points: OS (HR, 0.79; 95% CI, 0.45–1.40; *P* = 0.50), iDFS (HR, 0.81; 95% CI, 0.48–1.35; *P* = 0.50), RFS (HR, 0.80; 95% CI, 0.44–1.45; *P* = 0.54), and DRFi (HR, 0.77; 95% CI, 0.41–1.43; *P* = 0.48). Moreover, in high-risk group (Additional file 4: Figure S4B), the patients who received chemotherapy had an advantage of OS (HR, 2.38; 95% CI, 1.21–4.68; *P* = 0.04), and iDFS improvement (HR, 2.40; 95% CI, 1.31–4.38; *P* = 0.02) over patients received endocrine therapy alone; however, this survival advantage was not observed in either RFS (HR, 1.80; 95% CI, 0.97–3.34; *P* = 0.12) or DRFi (HR, 1.59; 95% CI, 0.85–2.96; *P* = 0.22). Our data showed chemotherapy may have benefits in CAB high-risk patients of exemestane arm alone, but due to small sample size, this cannot yet be convincingly shown.

## Discussion

Dramatically reduced mortality from breast cancer has been achieved due to the widespread application of adjuvant systemic therapy [17–20]. For women with early, HR-positive, HER2-negative breast cancer, adjuvant endocrine therapy is the cornerstone of systemic treatment, a few may also benefit from adjuvant chemotherapy. The magnitude of benefit from chemotherapy depends on both clinical features as well as tumor biology.

CAB, a prognostic test in early HR-positive HER2-negative breast cancer uses three clinical parameters and five distinctive biomarkers using an immunohistochemistry platform. Therefore, it would be much simpler to use and more economically efficient than pure gene-profiling-based prognostic tools [6, 16]. It is unique because this test uses immunohistochemistry combined with an SVML-based algorithm to predict risk scores [7, 21–25]. CAB segregates patients into two groups based on what corresponds to the CAB risk score with a cutoff of 15.5 [26–32]. Although the CAB has well-demonstrated data on cohorts from various geographies at five years since diagnosis, this is the first time we are showing the prognostic value of CAB at ten years of diagnosis in Dutch sub-cohort of the TEAM trial.

To validate a prognostic signature in a realistic manner, it is critical that the patient characteristics of the present study are diverse and distinct from those tested by previous prognostic tools. Most of the patients in our current study had clinical features of intermediate-recurrence risk, with about two-thirds involving one to three nodes, a larger  proportion of higher grade, nearly 80% of whom were TNM stage II and despite this approximately four-fifths of patients were treated with endocrine therapy alone. Therefore, the current study is interesting and important to understand how CAB would stratify these patients for this information to be used in daily clinical practice. Interestingly, the CAB risk score was confirmed to be an independent prognostic factor in this cohort of “intermediate clinical risk” regardless of tumor size, lymph node status and grade which shows the ‘importance’ of ‘tumor biology’ that CAB brings out over the clinical parameters.

There are a large proportion (about two-thirds) of patients stratified as low-risk, who had an 89% of DRFi at ten years, while only 70% of DRFi was achieved in the high-risk group, with an almost three-fold hazard of distant recurrence or death, which warrants the use of escalated therapies to reduce the risk of distant recurrence events in CAB high-risk group (Fig. 3B). Other clinicopathological factors were not statistically significant, which were consistent with previous findings from retrospective studies [13, 33].

A prognostic factor itself does not always have the value as a predictive factor. Although CAB could stratify patients in both randomized arms, CAB performed much better in the exemestane arm than the sequential arm, with much higher HR, and a highly significant *P*-value. It suggested patients with CAB low-risk receive exemestane rather than a sequential regimen to have a better DRFi (Fig. 3). The underlying mechanisms why CAB performs better in the exemestane arm needs further study. Previous exploratory analyses in the context of TEAM discovered several biomarkers or gene signatures that could predict the response of a sequential regimen [34–41]. These biomarkers studies continued in conjunction with CAB-based risk stratification can give us more insights into an endocrine response which will lead to better drugs in the years to come.

Although both MammaPrint and RS have been evaluated in node-positive disease, data for both are limited in this setting. In the RxPonder trial, postmenopausal women (two-thirds of those enrolled, nine percent  with three involved nodes) had over 90% of five-year invasive DFS without chemotherapy [42]. The follow-up has not been updated for up to ten years. In the PlanB study, for patients with low RS (approximately 34% of whom had N1 stage), the five-year disease-free survival was 95% [43, 44]. In this TEAM study, 67% of patients were with node-positive disease and CAB low-risk patients had 93% of DRFi at five years and this prognostic ability continues from five years to ten years from diagnosis which once again shows the usefulness of CAB in node-positive patients.

The absolute benefit of chemotherapy in patients with a low risk of recurrence is often small. The decision to prescribe chemotherapy must consider many parameters to avoid potential toxicities at best. Some experts strongly recommend offering chemotherapy to all patients with lymph node-positive disease. This approach may represent overtreatment for some patients who based on prognostic tests are at low risk. In this TEAM study, the patients who received exemestane alone, without chemotherapy, if CAB low-risk, had an excellent DRFi of 91% at ten years, irrespective of either node-negative or N1 stage. On the other hand, chemotherapy seemed to add survival benefits in CAB high-risk patients for OS and iDFS as endpoints. This is the first time that CAB has been shown to be predictive for use of chemotherapy in high-risk patients in a prospective randomized trial although with limited statistical power. The lack of chemotherapy benefit for DRFi endpoint in the total cohort is the limitation of this study. For patients with CAB high-risk who had low DRFi at ten years, other escalated treatments, for example, poly ADP ribose polymerase (PARP) inhibitors (when bearing BRCA mutations), CDK4/6 inhibitors, or a longer duration of endocrine therapy, etc. are options.

## Conclusions

In conclusion, in the Dutch sub-cohort of the TEAM trial, our findings revealed that CAB provides a prognostic signature for the clinical outcome for postmenopausal women with early breast cancer, HR-positive, HER2-negative disease of ten-year follow-up. It is very useful to help individualize endocrine-only treatment, for patients assigned to either the sequential endocrine regimen or exemestane alone.

## Supplementary Information

**Additional file 1: Fig. S1.** CONSORT flow diagram to account for missing patients in the Dutch sub-cohort of the TEAM trial. TNM stage IV was defined as evidence of metastatic disease. ^a^ Not applicated due to insufficient pathological slides. ^b^ This patient stopped adjuvant therapies and was lost of follow-up after receiving just two endocrine tablets due to serious adverse effects.

**Additional file 2: Fig. S2.** All-cause mortality, cumulative incidence of invasive breast cancer by CAB for the total cohort, and cumulative incidence of distant recurrence/death due to breast cancer (DM) by CAB for the subgroups. (A) All-cause mortality in the total cohort. (B) Cumulative incidence of invasive breast cancer for the entire cohort. (C) DM for patients who received adjuvant exemestane alone. (D) DM for patients who received sequential adjuvant regimen alone.

**Additional file 3: Fig. S3.** DM by CAB in subgroups. (A) DM at five years in lymph node-positive (N+) patients. (B) N+ patients in the exemestane arm. (C) N+ patients in the sequential arm.

**Additional file 4: Fig. S4.** Hazard ratio estimates of overall survival (OS), invasive disease-free survival (iDFS), relapse-free survival (RFS), and distant recurrence-free interval (DRFi) between patients with or without chemotherapy or randomized arms receiving endocrine therapy alone in two CanAssist Breast (CAB) risk categories. (A) Low-risk group. (B) High-risk group.

## Data Availability

The datasets used and analyzed during the current study are available from the corresponding authors on reasonable request.

## Abbreviations

HR: Hormone receptor
HER2: Human epidermal growth factor receptor 2
TEAM: Tamoxifen, Exemestane Adjuvant Multinational clinical trial
CAB: CanAssist Breast
HRs: Hazard ratios
DM: Cumulative incidence of distant metastasis/death due to breast cancer
DRFi: Distant recurrence-free interval
RFS: Recurrence-free survival
OS: Overall survival
iDFS: Invasive disease-recurrent free survival
IHC: Immunohistochemistry
SVML: Support Vector Machine Learning

## References

[1] Giorgi Rossi P, Lebeau A, Canelo-Aybar C, Saz-Parkinson Z, Quinn C, Langendam M, McGarrigle H, Warman S, Rigau D, Alonso-Coello P (2021). Recommendations from the European Commission Initiative on Breast Cancer for multigene testing to guide the use of adjuvant chemotherapy in patients with early breast cancer, hormone receptor positive, HER-2 negative. Br J Cancer.

[2] Goncalves R, Bose R (2013). Using multigene tests to select treatment for early-stage breast cancer. J Natl Compr Canc Netw.

[3] Sparano JA, Paik S (2008). Development of the 21-gene assay and its application in clinical practice and clinical trials. J Clin Oncol.

[4] Paik S, Shak S, Tang G, Kim C, Baker J, Cronin M, Baehner FL, Walker MG, Watson D, Park T (2004). A multigene assay to predict recurrence of tamoxifen-treated, node-negative breast cancer. N Engl J Med.

[5] Troester MA, Sun X, Allott EH, Geradts J, Cohen SM, Tse CK, Kirk EL, Thorne LB, Mathews M, Li Y (2018). Racial differences in PAM50 subtypes in the Carolina Breast Cancer Study. J Natl Cancer Inst.

[6] Serkad CPV, Attuluri AK, Basavaraj C, Adinarayan M, Krishnamoorthy N, Ananthamurthy SB, Mallikarjuna SE, Bakre MM (2021). Validation of CanAssist Breast immunohistochemistry biomarkers on an automated platform and its applicability in tissue microarray. Int J Clin Exp Pathol.

[7] Parikh PM, Bhattacharyya GS, Biswas G, Krishnamurty A, Doval D, Heroor A, Sharma S, Deshpande R, Chaturvedi H, Somashekhar SP (2021). Practical consensus recommendations for optimizing risk versus benefit of chemotherapy in patients with HR positive Her2 negative early breast cancer in India. South Asian J Cancer.

[8] Chandra Doval D, Mehta A, Somashekhar SP, Gunda A, Singh G, Bal A, Khare S, Prakash VSC, Adinarayan M, Krishnamoorthy N (2021). The usefulness of CanAssist breast in the assessment of recurrence risk in patients of ethnic Indian origin. Breast.

[9] Sankaran S, Dikshit JB, Prakash Sv C, Mallikarjuna SE, Somashekhar SP, Patil S, Kumar R, Prasad K, Shet D, Bakre MM (2021). CanAssist breast impacting clinical treatment decisions in early-stage HR+ breast cancer patients: Indian Scenario. Indian J Surg Oncol.

[10] Derks MGM, Blok EJ, Seynaeve C, Nortier JWR, Kranenbarg EM, Liefers GJ, Putter H, Kroep JR, Rea D, Hasenburg A (2017). Adjuvant tamoxifen and exemestane in women with postmenopausal early breast cancer (TEAM): 10-year follow-up of a multicentre, open-label, randomised, phase 3 trial. Lancet Oncol.

[11] Gunda A, Basavaraj C, Serkad VC, Adinarayan M, Kolli R, Siraganahalli Eshwaraiah M, Saura C, Ruiz F, Gomez P, Peg V (2022). A retrospective validation of CanAssist Breast in European early-stage breast cancer patient cohort. Breast.

[12] Sengupta AK, Gunda A, Malpani S, Serkad CPV, Basavaraj C, Bapat A, Bakre MM (2020). Comparison of breast cancer prognostic tests CanAssist Breast and Oncotype DX. Cancer Med.

[13] Bakre MM, Ramkumar C, Attuluri AK, Basavaraj C, Prakash C, Buturovic L, Madhav L, Naidu N, Prathima R, Somashekhar SP (2019). Clinical validation of an immunohistochemistry-based CanAssist-Breast test for distant recurrence prediction in hormone receptor-positive breast cancer patients. Cancer Med.

[14] van de Velde CJ, Rea D, Seynaeve C, Putter H, Hasenburg A, Vannetzel JM, Paridaens R, Markopoulos C, Hozumi Y, Hille ET (2011). Adjuvant tamoxifen and exemestane in early breast cancer (TEAM): a randomised phase 3 trial. Lancet.

[15] Martinez Guisado A, Sanchez Munoz A, de la Cabeza Lomas Garrido M, Ruiz Borrego M, Bayo Calero J, de la Toro Salas R, Gonzalez Mancha R, de Haba Rodriguez J, Alba Conejo E (2011). Initialization of adjuvant hormonal treatment for breast cancer. Adv Ther.

[16] Ramkumar C, Buturovic L, Malpani S, Kumar Attuluri A, Basavaraj C, Prakash C, Madhav L, Doval DC, Mehta A, Bakre MM (2018). Development of a novel proteomic risk-classifier for prognostication of patients with early-stage hormone receptor-positive breast cancer. Biomark Insights.

[17] Early Breast Cancer Trialists' Collaborative G, Peto R, Davies C, Godwin J, Gray R, Pan HC, Clarke M, Cutter D, Darby S, McGale P et al. Comparisons between different polychemotherapy regimens for early breast cancer: meta-analyses of long-term outcome among 100,000 women in 123 randomised trials. Lancet 2012;379(9814):432–44. 10.1016/S0140-6736(11)61625-5 PMC3273723 22152853

[18] Early Breast Cancer Trialists' Collaborative G, Davies C, Godwin J, Gray R, Clarke M, Cutter D, Darby S, McGale P, Pan HC, Taylor C et al. Relevance of breast cancer hormone receptors and other factors to the efficacy of adjuvant tamoxifen: patient-level meta-analysis of randomised trials. Lancet 2011;378(9793):771–84. 10.1016/S0140-6736(11)60993-8 PMC3163848 21802721

[19] Early Breast Cancer Trialists' Collaborative G, Darby S, McGale P, Correa C, Taylor C, Arriagada R, Clarke M, Cutter D, Davies C, Ewertz M et al. Effect of radiotherapy after breast-conserving surgery on 10-year recurrence and 15-year breast cancer death: meta-analysis of individual patient data for 10,801 women in 17 randomised trials. Lancet 2011;378(9804):1707–16. 10.1016/S0140-6736(11)61629-2 PMC3254252 22019144

[20] Forouzanfar MH, Foreman KJ, Delossantos AM, Lozano R, Lopez AD, Murray CJ, Naghavi M (2011). Breast and cervical cancer in 187 countries between 1980 and 2010: a systematic analysis. Lancet.

[21] Bagchee-Clark AJ, Mucaki EJ, Whitehead T, Rogan PK (2020). Pathway-extended gene expression signatures integrate novel biomarkers that improve predictions of patient responses to kinase inhibitors. MedComm.

[22] Nguyen LC, Naulaerts S, Bruna A, Ghislat G, Ballester PJ (2021). Predicting cancer drug response in vivo by learning an optimal feature selection of tumour molecular profiles. Biomedicines.

[23] Vergara HM, Pape C, Meechan KI, Zinchenko V, Genoud C, Wanner AA, Mutemi KN, Titze B, Templin RM, Bertucci PY (2021). Whole-body integration of gene expression and single-cell morphology. Cell.

[24] Somepalli G, Sahoo S, Singh A, Hannenhalli S (2021). Prioritizing and characterizing functionally relevant genes across human tissues. PLoS Comput Biol.

[25] Assiri AS, Nazir S, Velastin SA (2020). Breast tumor classification using an ensemble machine learning method. J Imaging.

[26] Hardt O, Wild S, Oerlecke I, Hofmann K, Luo S, Wiencek Y, Kantelhardt E, Vess C, Smith GP, Schroth GP (2012). Highly sensitive profiling of CD44+/CD24− breast cancer stem cells by combining global mRNA amplification and next generation sequencing: evidence for a hyperactive PI3K pathway. Cancer Lett.

[27] Qiu Y, Pu T, Guo P, Wei B, Zhang Z, Zhang H, Zhong X, Zheng H, Chen L, Bu H (2016). ALDH(+)/CD44(+) cells in breast cancer are associated with worse prognosis and poor clinical outcome. Exp Mol Pathol.

[28] Inoue K, Fry EA (2015). Aberrant splicing of estrogen receptor, HER2, and CD44 genes in breast cancer. Genet Epigenet.

[29] Kozawa K, Sekai M, Ohba K, Ito S, Sako H, Maruyama T, Kakeno M, Shirai T, Kuromiya K, Kamasaki T (2021). The CD44/COL17A1 pathway promotes the formation of multilayered, transformed epithelia. Curr Biol.

[30] Sharma M, Mittapelly N, Banala VT, Urandur S, Gautam S, Marwaha D, Rai N, Singh N, Gupta A, Mitra K et al. Amalgamated microneedle array bearing ribociclib-loaded transfersomes eradicates breast cancer via CD44 targeting. Biomacromolecules 2022;23(3):661–675. 10.1021/acs.biomac.1c01076 34978424

[31] Islam MS, Islam MS, Parvin S, Ahmed MU, Bin Sayeed MS, Uddin MM, Hussain SM, Hasnat A (2015). Effect of GSTP1 and ABCC4 gene polymorphisms on response and toxicity of cyclophosphamide-epirubicin-5-fluorouracil-based chemotherapy in Bangladeshi breast cancer patients. Tumour Biol.

[32] Uwai Y (2018). Enantioselective drug recognition by drug transporters. Molecules.

[33] Attuluri AK, Serkad CPV, Gunda A, Ramkumar C, Basavaraj C, Buturovic L, Madhav L, Naidu N, Krishnamurthy N, Prathima R (2019). Analytical validation of CanAssist-Breast: an immunohistochemistry based prognostic test for hormone receptor positive breast cancer patients. BMC Cancer.

[34] Bartlett JMS, Ahmed I, Regan MM, Sestak I, Mallon EA, Dell'Orto P, Thurlimann B, Seynaeve C, Putter H, Van de Velde CJH (2017). HER2 status predicts for upfront AI benefit: A TRANS-AIOG meta-analysis of 12,129 patients from ATAC, BIG 1–98 and TEAM with centrally determined HER2. Eur J Cancer.

[35] Fontein DBY, Klinten Grand M, Nortier JWR, Seynaeve C, Meershoek-Klein Kranenbarg E, Dirix LY, van de Velde CJH, Putter H (2015). Dynamic prediction in breast cancer: proving feasibility in clinical practice using the TEAM trial. Ann Oncol.

[36] Noordhoek I, Blok EJ, Meershoek-Klein Kranenbarg E, Putter H, Duijm-de Carpentier M, Rutgers EJT, Seynaeve C, Bartlett JMS, Vannetzel JM, Rea DW (2020). Overestimation of late distant recurrences in high-risk patients with ER-positive breast cancer: validity and accuracy of the CTS5 risk score in the TEAM and IDEAL trials. J Clin Oncol.

[37] Engels CC, de Glas NA, Sajet A, Bastiaannet E, Smit VT, Kuppen PJ, Seynaeve C, van de Velde CJ, Liefers GJ (2016). The influence of insulin-like Growth Factor-1-Receptor expression and endocrine treatment on clinical outcome of postmenopausal hormone receptor positive breast cancer patients: a Dutch TEAM substudy analysis. Mol Oncol.

[38] Bartlett JM, Brookes CL, Piper T, van de Velde CJ, Stocken D, Lyttle N, Hasenburg A, Quintayo MA, Kieback DG, Putter H (2013). Do type 1 receptor tyrosine kinases inform treatment choice? A prospectively planned analysis of the TEAM trial. Br J Cancer.

[39] Bartlett JM, Christiansen J, Gustavson M, Rimm DL, Piper T, van de Velde CJ, Hasenburg A, Kieback DG, Putter H, Markopoulos CJ (2016). Validation of the IHC4 breast cancer prognostic algorithm using multiple approaches on the multinational TEAM clinical trial. Arch Pathol Lab Med.

[40] Roseweir AK, Bennett L, Dickson A, Cheng K, Quintayo MA, Bayani J, McMillan DC, Horgan PG, van de Velde CJH, Seynaeve C (2018). Predictive biomarkers for endocrine therapy: retrospective study in tamoxifen and exemestane adjuvant multinational (TEAM) trial. J Natl Cancer Inst.

[41] Engels CC, Charehbili A, van de Velde CJ, Bastiaannet E, Sajet A, Putter H, van Vliet EA, van Vlierberghe RL, Smit VT, Bartlett JM (2015). The prognostic and predictive value of Tregs and tumor immune subtypes in postmenopausal, hormone receptor-positive breast cancer patients treated with adjuvant endocrine therapy: a Dutch TEAM study analysis. Breast Cancer Res Treat.

[42] Kalinsky K, Barlow WE, Gralow JR, Meric-Bernstam F, Albain KS, Hayes DF, Lin NU, Perez EA, Goldstein LJ, Chia SKL (2021). 21-Gene assay to inform chemotherapy benefit in node-positive breast cancer. N Engl J Med.

[43] Gluz O, Nitz UA, Christgen M, Kates RE, Shak S, Clemens M, Kraemer S, Aktas B, Kuemmel S, Reimer T (2016). West German Study Group Phase III PlanB trial: first prospective outcome data for the 21-gene recurrence score assay and concordance of prognostic markers by central and local pathology assessment. J Clin Oncol.

[44] Nitz U, Gluz O, Clemens M, Malter W, Reimer T, Nuding B, Aktas B, Stefek A, Pollmanns A, Lorenz-Salehi F (2019). West German Study PlanB trial: adjuvant four cycles of epirubicin and cyclophosphamide plus docetaxel versus six cycles of docetaxel and cyclophosphamide in HER2-negative early breast cancer. J Clin Oncol.

