# Supplementary figures and images for: Ten-year distant-recurrence risk prediction in breast cancer by CanAssist Breast (CAB) in Dutch sub-cohort of the randomized TEAM trial
Source: Breast Cancer Res. 2023 Apr 14;25:40. doi: 10.1186/s13058-023-01643-2 (PMC10103430; doi:10.1186/s13058-023-01643-2)

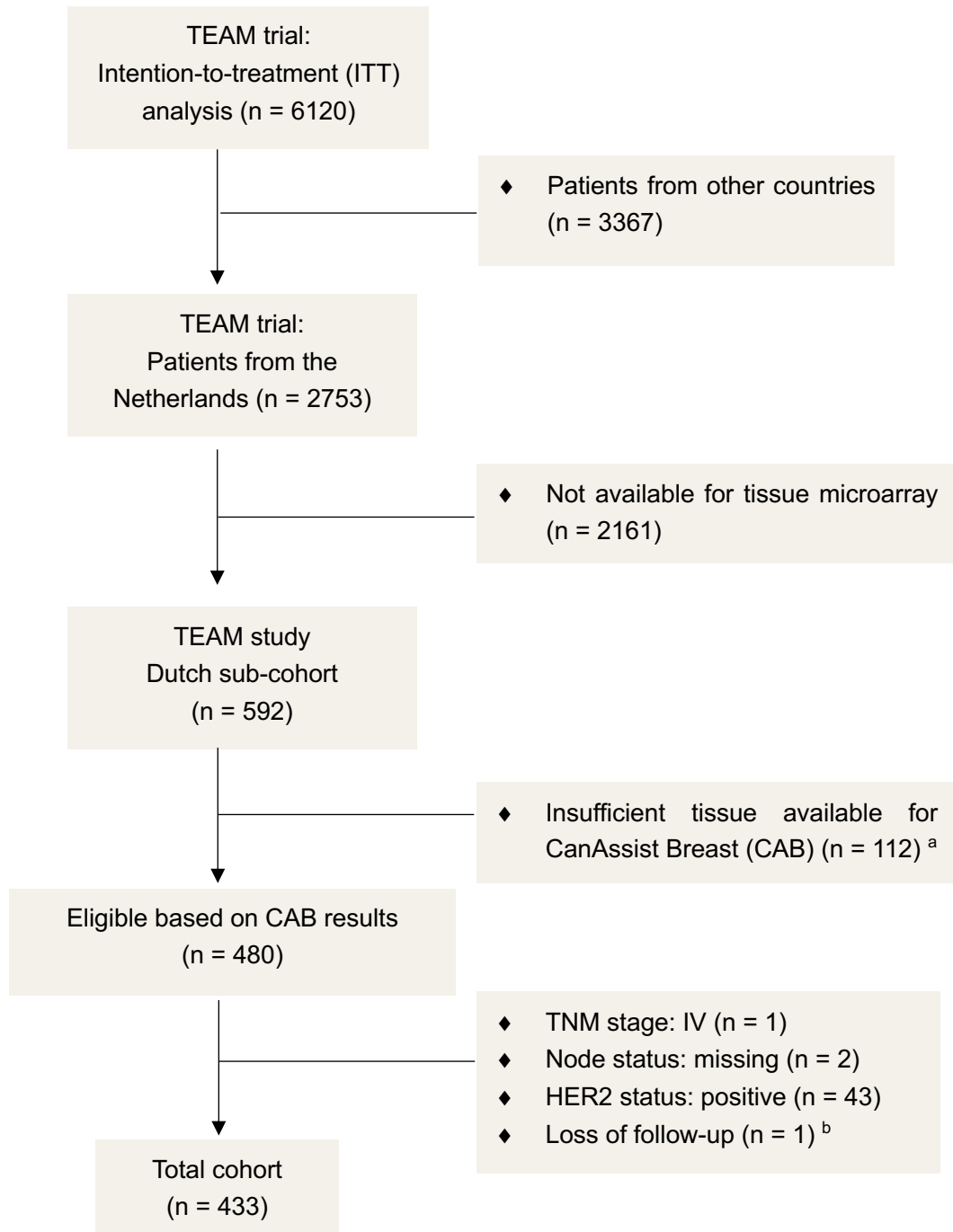

Supplement: Supplementary file 1 — Additional file 1: Fig. S1. CONSORT flow diagram to account for missing patients in the Dutch sub-cohort of the TEAM trial. TNM stage IV was defined as evidence of metastatic disease. a Not applicated due to insufficient pathological slides. b This patient stopped adjuvant therapies and was lost of follow-up after receiving just two endocrine tablets due to serious adverse effects. [file 13058_2023_1643_MOESM1_ESM.pdf]

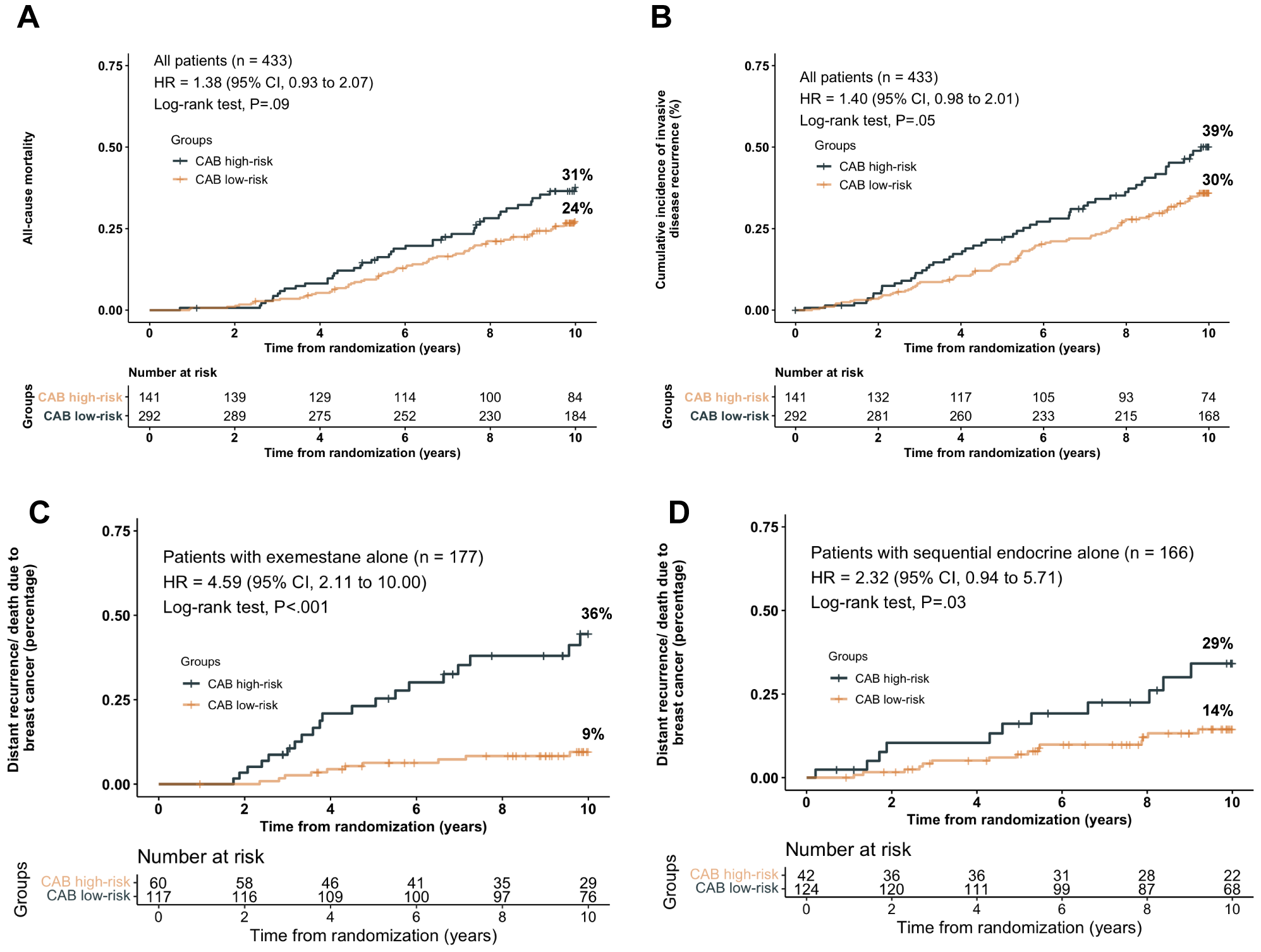

Supplement: Supplementary file 2 — Additional file 2: Fig. S2. All-cause mortality, cumulative incidence of invasive breast cancer by CAB for the total cohort, and cumulative incidence of distant recurrence/death due to breast cancer (DM) by CAB for the subgroups. (A) All-cause mortality in the total cohort. (B) Cumulative incidence of invasive breast cancer for the entire cohort. (C) DM for patients who received adjuvant exemestane alone. (D) DM for patients who received sequential adjuvant regimen alone. [file 13058_2023_1643_MOESM2_ESM.tif]

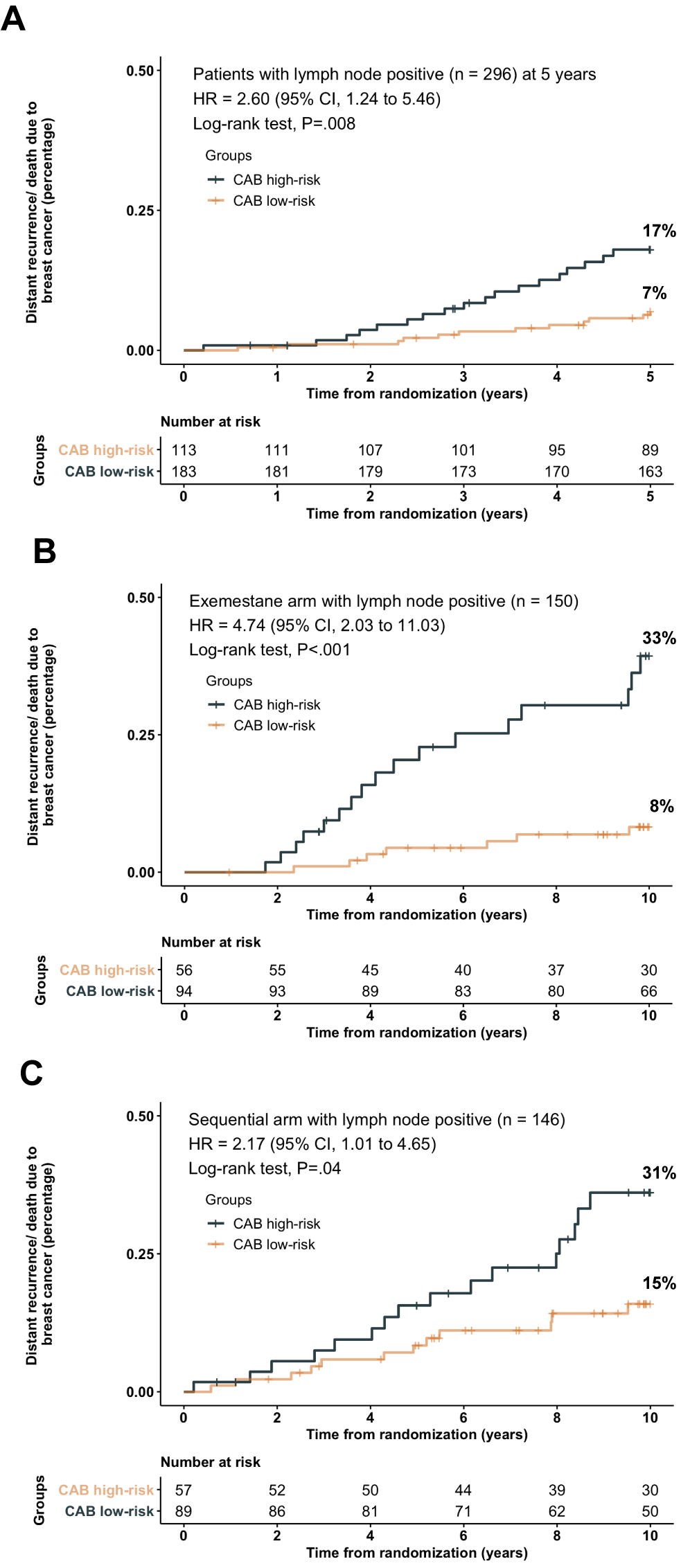

Supplement: Supplementary file 3 — Additional file 3: Fig. S3. DM by CAB in subgroups. (A) DM at five years in lymph node-positive (N+) patients. (B) N+ patients in the exemestane arm. (C) N+ patients in the sequential arm. [file 13058_2023_1643_MOESM3_ESM.tif]

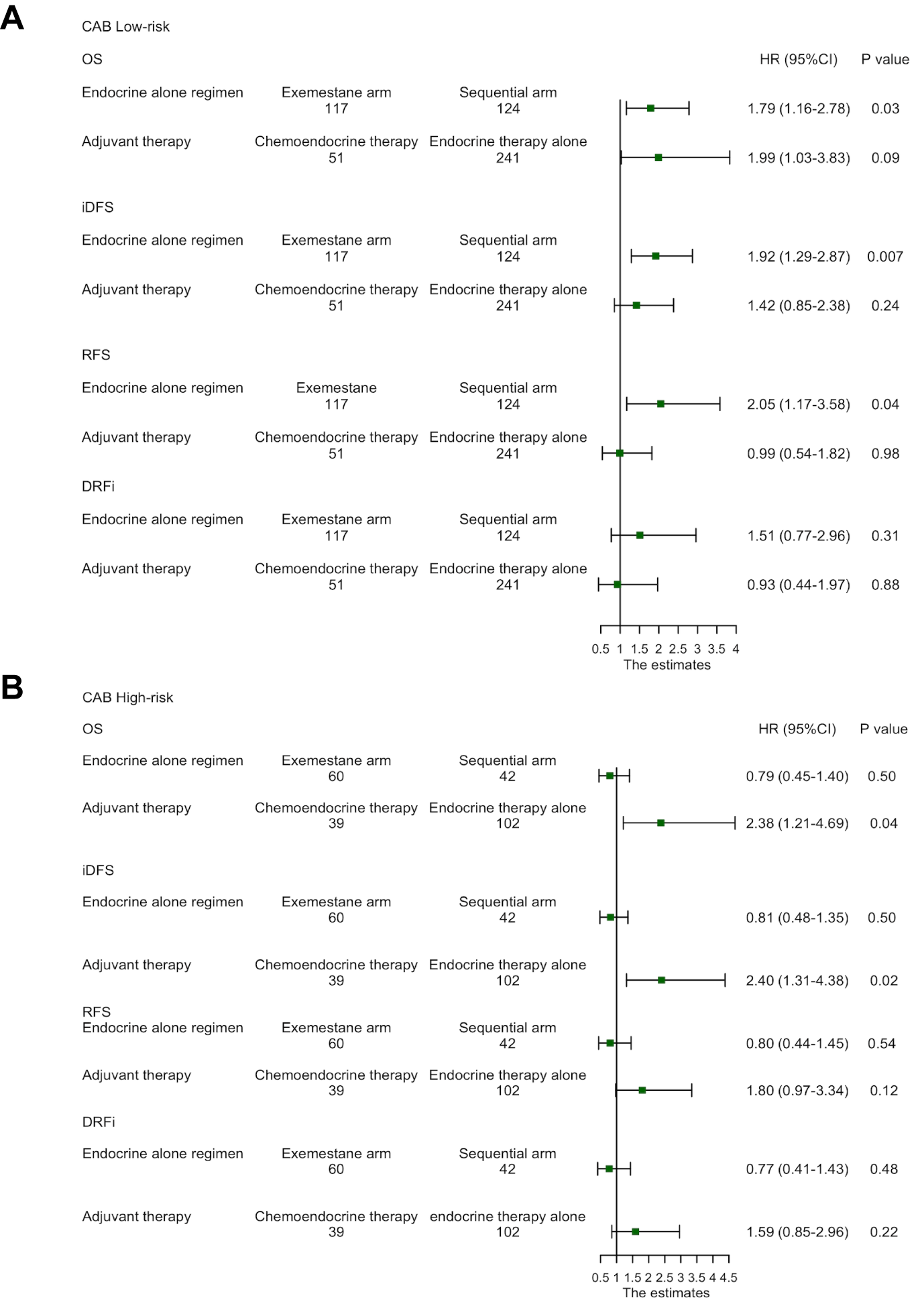

Supplement: Supplementary file 4 — Additional file 4: Fig. S4. Hazard ratio estimates of overall survival (OS), invasive disease-free survival (iDFS), relapse-free survival (RFS), and distant recurrence-free interval (DRFi) between patients with or without chemotherapy or randomized arms receiving endocrine therapy alone in two CanAssist Breast (CAB) risk categories. (A) Low-risk group. (B) High-risk group. [file 13058_2023_1643_MOESM4_ESM.tif]
